# Supplementary material for: COPI mediates recycling of an exocytic SNARE by recognition of a ubiquitin sorting signal
Source: eLife. 2017 Oct 23;6:e28342. doi: 10.7554/eLife.28342 (PMC5663479; doi:10.7554/eLife.28342)
Supplement: Supplementary file 1. [file elife-28342-supp1.docx]

List of plasmids used in this study

| **Name** | **Plasmids** | **Description** | **Sources** |
| --- | --- | --- | --- |
| Vector | pRS416 | Yeast shuttle vector | ATCC |
| GFP-Snc1 WT | pRS146-GFP-Snc1 | GFP-tagged Snc1 | ([Lewis et al., 2000](#_ENREF_30)) |
| DUB-GFP-Snc1 | pRS146-DUB-GFP-Snc1 | Deubiquitinase(UL36) catalytic domain tagged GFP-Snc1 | This study |
| DUB*-GFP-Snc1 | pRS146-DUB*-GFP-Snc1 | Catalytic dead form of deubiquitinase tagged GFP-Snc1 | This study |
| GFP-Snc1-PM | pRS416-GFP-Snc1-PM | GFP tagged Snc1 with mutations in the Snc1 endocytosis signal | This study |
| DUB-GFP-Snc1-PM | pRS416-DUB-GFP-Snc1-PM | Deubiquitinase tagged GFP-Snc1-PM | This study |
| mCherry-Tlg1 | pRS315-mCherry-Tlg1 | mCherry tagged Tlg1 | ([Xu et al., 2013](#_ENREF_64)) |
| 3xHA-Snc1 WT | pRS416-3xHA-Snc1 | 3xHA tagged Snc1 | This study |
| 3xHA-Snc1 8KR | pRS416-3xHA-Snc1 8KR | 3xHA tagged lysine-less Snc1 | This study |
| 3xFLAG-Ub | pRS315-3xFLAG-Ub | 3xFLAG tagged Ub gene | This study |
| ss-Myc-Emp47 | pRS416-ss-Myc-EMP47 | N-terminal Myc tagged EMP47 after signal sequence cleavage site | This study |
| β’-COP WT | pRS315-SEC27 | *SEC27* (β'-COP) whole coding cassette including its promoter and terminator | This study |
| β'-COP RKR | pRS315-sec27 RKR | *SEC27* dilysine binding site mutant | This study |
| β'-COP Δ2-304 | pRS315-sec27Δ2-304 | *SEC27* with deletion the N-terminal β-propeller | This study |
| β'-COP human beta-Propeller | pRS315-hCOPB2(1-303)-sec(305-899) | *SEC27* first β-propeller replaced with human β'-COP propeller | This study |
| β'-COP UBD_Doa1_ | pRS315-Doa1(1-450)-Sec27(305-889) | *SEC27* first β-propeller replaced with DOA1(1-450) | This study |
| β'-COP NZF_Tab2_ | pRS315-NZF-Sec27(305-899) | *SEC27* first β-propeller replaced with NZF (TAB2 aa 665-693) | This study |
| α-COP WT | pRS313-COP1 | COP1 whole coding cassette including its promoter and terminator | This study |
| α-COP Δ2-324 | pRS313-cop1(325-1201) | COP1 deleting the first β-propeller | This study |
| human beta-Propeller-GST | pCOPB2(1-303)-GST | human β'-COP N-terminal propeller tagged with GST | This study |
| GFP-Rer1 | pRS416-GFP-Rer1 | GFP-tagged Rer1 | ([Sato et al., 1995](#_ENREF_46)) |
| Pib1-DUB | pRS416-CUP1-Pib1-DUB | DUB tagged Pib1 | ([MacDonald et al., 2017](#_ENREF_33)) |
| Tul1-DUB | pRS416-CUP1-Tul1-DUB | DUB tagged Tul1 | ([MacDonald et al., 2017](#_ENREF_33)) |
| Vps11-DUB | pRS416-CUP1-Vps11-DUB | DUB tagged Vps11 | ([MacDonald et al., 2017](#_ENREF_33)) |
| Rcy1-DUB | pRS416-CUP1-Rcy1-DUB | DUB tagged Rcy1 | ([MacDonald et al., 2017](#_ENREF_33)) |
| Rsp5-DUB | pRS416-CUP1-Rsp5-DUB | DUB tagged Rsp5 | ([MacDonald et al., 2017](#_ENREF_33)) |
| Pib1-DUB | pRS416-CUP1-Pib1-DUB* | DUB dead mutant tagged Pib1 | ([MacDonald et al., 2017](#_ENREF_33)) |
| Tul1-DUB | pRS416-CUP1-Tul1-DUB* | DUB dead mutant tagged Tul1 | ([MacDonald et al., 2017](#_ENREF_33)) |
| DUB-Rcy1 | pRS313-DUB-Rcy1 | DUB tagged Rcy1 in its N-terminus with Rcy1 promoter | This study |
| DUB*-Rcy1 | pRS313-DUB*-Rcy1 | DUB dead mutant tagged Rcy1 in its N-terminus with Rcy1 promoter | This study |
| Ina1-mNeonGreen | pRS416-ADH-Ina1-mNeonGreen | mNeonGreen-tagged plasma membrane protein Ina1 | This study |
| Tat1-mNeonGreen | pRS416-ADH-Ina1-mNeonGreen | mNeonGreen-tagged plasma membrane protein Tat1 | This study |

References

Lewis, M.J., B.J. Nichols, C. Prescianotto-Baschong, H. Riezman, and H.R. Pelham. 2000. Specific retrieval of the exocytic SNARE Snc1p from early yeast endosomes. *Mol Biol Cell*. 11:23-38.

Xu, P., R.D. Baldridge, R.J. Chi, C.G. Burd, and T.R. Graham. 2013. Phosphatidylserine flipping enhances membrane curvature and negative charge required for vesicular transport. *J Cell Biol*. 202:875-886.

Sato, K., S. Nishikawa, and A. Nakano. 1995. Membrane protein retrieval from the Golgi apparatus to the endoplasmic reticulum (ER): characterization of the RER1 gene product as a component involved in ER localization of Sec12p. *Molecular biology of the cell*. 6:1459-1477.

MacDonald, C., S. Winistorfer, R.M. Pope, M.E. Wright, and R.C. Piper. 2017. Enzyme reversal to explore the function of yeast E3 ubiquitin-ligases. *Traffic*.
