# Supplementary material for: COPI mediates recycling of an exocytic SNARE by recognition of a ubiquitin sorting signal
Source: eLife. 2017 Oct 23;6:e28342. doi: 10.7554/eLife.28342 (PMC5663479; doi:10.7554/eLife.28342)
Supplement: Supplementary file 2. [file elife-28342-supp2.docx]

List of strains used in this study

| **Name** | **Genotype** | **Annotation** | **Sources** |
| --- | --- | --- | --- |
| BY4742 | *MATα his3 leu2 ura3 lys2* | WT | Invitrogen |
| BY4742 YJL204C | *MATα his3 leu2 ura3 lys2 Rcy1::KanMX6* | RCY1 knockout | Invitrogen |
| ZHY615M2D | *MATα his3 leu2 ura3 lys2 drs2∆::Kan* | DRS2 knockout | ([Hua et al., 2002](#_ENREF_5)) |
| PXY46 | *MATα his3 leu2 ura3 lys2 DRS2::UL36-3xHA::ClonNAT* | DRS2-DUB | This study |
| PXY47 | *MATα his3 leu2 ura3 lys2 DRS2::UL36*-3xHA::ClonNAT* | DRS2-DUB C57S deadmutant | This study |
| KLY691 | *Mata his3 leu2 ura3 gga1∆::KanMX6 gga2∆::KanMX6* | GGA pathway mutant | Invitrogen |
| BY4742 YPR029C | *MATα his3 leu2 ura3 lys2 apl4∆::KanMX6* | AP1 pathway mutant | Invitrogen |
| BY4742 YPL195W | *MATα his3 leu2 ura3 lys2 apl5∆::KanMX6* | AP3 pathway mutant | Invitrogen |
| BY4742 YJR058C | *MATα his3 leu2 ura3 lys2 aps2∆::KanMX7* | AP2 pathway mutant | Invitrogen |
| EGY101-16d | *MATa ret1-1 leu2-3,112 ura3-52 his3-∆200 trp1-∆901 suc2-∆9* | COP1 temperature sensitive mutant | (Gaynor and Emr, 1997) |
| PXY2174A | *MATα his3 leu2 ura3 lys2 sec27Δ::Hygro p315-SEC27* | SEC27 whole coding cassette | This study |
| PXY2175A | *MATα his3 leu2 ura3 lys2 sec27Δ::Hygro p315-sec27Δ2-304* | SEC27 deleting the first beta-Propeller | This study |
| PXY2186A | *MATα his3 leu2 ura3 lys2 sec27Δ::Hygro p315-sec27 RKR* | SEC27 dilysine binding site mutant | This study |
| PXY2193A | *MATα his3 leu2 ura3 lys2 sec27Δ::Hygro p315-hCOPB2(1-303)-sec(305-899)* | SEC27 first propeller replaced with human beta'-COP propeller | This study |
| PXY2184A | *MATα his3 leu2 ura3 lys2 sec27Δ::Hygro p315-Doa1(1-450)-Sec27(305-889)* | SEC27 the first beta-propeller replaced with DOA1(1-450) | This study |
| PXY2192A | *MATα his3 leu2 ura3 lys2 sec27Δ::Hygro p315-NZF-Sec27(305-899)* | SEC27 the first beta-Propeller replaced with NZF (TAB2 aa 665-693) | This study |
| PXY2198A | *MATα his3 leu2 ura3 lys2 cop1Δ::Hygro p313-COP1* | COP1 whole coding cassette | This study |
| PXY2199A | *MATα his3 leu2 ura3 lys2 cop1Δ::Hygro p313-cop1(325-1201)* | COP1 deleting the first beta-propeller | This study |
| PXY2100A | *BY4742 Cop1::mKate p313-GFP-Rer1* |  | This study |
| PXY2101A | *BY4742 Cop1::mKate p315-GFP-Tlg1* |  | This study |
| PXY2101C | *BY4742 Cop1::mKate p315-GFP-Tlg1* |  | This study |
| PXY2102A | *BY4742 Cop1::GFP::HIS3 Sec7::mKate::URA* |  | This study |
| PXY2103A | *BY4742 p416-GFP-Rer1 p313-mCherry-Tlg1* |  | This study |
| PLY5293 | *BY4742 pib1∆::KanMX6* | PIB1 knockout | Invitrogen |
| PLY5294 | *BY4742 tul1∆::KanMX6* | TUL1 knockout | Invitrogen |
| PXY64 | *BY4742 pib1∆::KanMX6 tul1∆::mtx* | PIB1TUL1 double knockout | This study |

References

Hua, Z., P. Fatheddin, and T.R. Graham. 2002. An essential subfamily of Drs2p-related P-type ATPases is required for protein trafficking between Golgi complex and endosomal/vacuolar system. *Mol Biol Cell*. 13:3162-3177.

Gaynor, E.C., and S.D. Emr. 1997. COPI-independent anterograde transport: cargo-selective ER to Golgi protein transport in yeast COPI mutants. *The Journal of cell biology*. 136:789-802.
