# Supplementary material for: COPI mediates recycling of an exocytic SNARE by recognition of a ubiquitin sorting signal
Source: eLife. 2017 Oct 23;6:e28342. doi: 10.7554/eLife.28342 (PMC5663479; doi:10.7554/eLife.28342)
Supplement: Transparent reporting form [file elife-28342-transrepform.docx]

***eLife’s* transparent reporting form**

We encourage authors to provide detailed information *within their submission* to facilitate the interpretation and replication of experiments. If you have any questions, please contact us: [editorial@elifesciences.org](mailto:editorial@elifesciences.org).

**Sample-size estimation**

- You should state whether an appropriate sample size was computed when the study was being designed
- You should state the statistical method of sample size computation and any required assumptions
- If no explicit power analysis was used, you should describe how you decided what sample (replicate) size (number) to use

Please outline where this information can be found within the submission (e.g., page numbers or figure legends), or explain why this information doesn’t apply to your submission:

For cell imaging work, we routinely use 3 biological replicates for each genotype and each replicate includes data from 50 cells for the quantitation. Representative images from the 150 cells quantified were chosen for presentation and all microscopy was quantified. Protein interaction experiments were quantified from at least three independent experiments. We state the sample size and statistical methods in each legend. Methods for quantifying images and immunoblots are described in either the legends or the methods section.

**Replicates**

- You should report how often each experiment was performed
- You should include a definition of biological versus technical replication
- The data obtained should be provided and sufficient information should be provided to indicate the number of independent biological and/or technical replicates
- If you encountered any outliers, you should describe how these were handled
- Criteria for exclusion/inclusion of data should be clearly stated
- High-throughput sequence data should be uploaded before submission, with a private link for reviewers provided (these are available from both GEO and ArrayExpress)

Please outline where this information can be found within the submission (e.g., page numbers or figure legends), or explain why this information doesn’t apply to your submission:

Biological replicates are defined as independently isolated yeast strains with the same genotype. All information on replicates can be found in figure legend and Methods. The pull-down experiments were performed with at least two separate batches of GST-fusion proteins. Replicates were independently performed binding reactions that were performed multiple times (~10 times) to work out conditions for the assays. Data shown were averages from three independent experiments once the final conditions were chosen.

**Statistical reporting**

- Statistical analysis methods should be described and justified
- Raw data should be presented in figures whenever informative to do so (typically when N per group is less than 10)
- For each experiment, you should identify the statistical tests used, exact values of N, definitions of center, methods of multiple test correction, and dispersion and precision measures (e.g., mean, median, SD, SEM, confidence intervals; and, for the major substantive results, a measure of effect size (e.g., Pearson's r, Cohen's d)
- Report exact p-values wherever possible alongside the summary statistics and 95% confidence intervals. These should be reported for all key questions and not only when the p-value is less than 0.05.

We present all of statistical analysis described in the legends and Methods. All of the statistical analyses of the data can be found in the uploaded table.

Please outline where this information can be found within the submission (e.g., page numbers or figure legends), or explain why this information doesn’t apply to your submission:

(For large datasets, or papers with a very large number of statistical tests, you may upload a single table file with tests, Ns, etc., with reference to page numbers in the manuscript.)

**Additional data files (“source data”)**

- We encourage you to upload relevant additional data files, such as numerical data that are represented as a graph in a figure, or as a summary table
- Where provided, these should be in the most useful format, and they can be uploaded as “Source data” files linked to a main figure or table
- Include model definition files including the full list of parameters used
- Include code used for data analysis (e.g., R, MatLab)
- Avoid stating that data files are “available upon request”

Please indicate the figures or tables for which source data files have been provided:

We will upload all of statistical data in excel files.
